# The Causes and Consequences of Spatial Organization of the Genome in Regulation of Gene Expression

**DOI:** 10.3389/fimmu.2021.682397

**Published:** 2021-06-04

**Authors:** Marios Agelopoulos, Spyros Foutadakis, Dimitris Thanos

**Affiliations:** Biomedical Research Foundation, Academy of Athens, Athens, Greece

**Keywords:** regulation of transcription, enhancers, transcription factors, chromatin, enhancer hubs, stochastic expression

## Abstract

Regulation of gene expression in time, space and quantity is orchestrated by the functional interplay of cis-acting elements and trans-acting factors. Our current view postulates that transcription factors recognize enhancer DNA and read the transcriptional regulatory code by cooperative DNA binding to specific DNA motifs, thus instructing the recruitment of transcriptional regulatory complexes forming a plethora of higher-ordered multi-protein-DNA and protein-protein complexes. Here, we reviewed the formation of multi-dimensional chromatin assemblies implicated in gene expression with emphasis on the regulatory role of enhancer hubs as coordinators of stochastic gene expression. Enhancer hubs contain many interacting regulatory elements and represent a remarkably dynamic and heterogeneous network of multivalent interactions. A functional consequence of such complex interaction networks could be that individual enhancers function synergistically to ensure coordination, tight control and robustness in regulation of expression of spatially connected genes. In this review, we discuss fundamental paradigms of such inter- and intra- chromosomal associations both in the context of immune-related genes and beyond.

## What Are the Enhancers

Regulation of gene expression in time, space and quantity is orchestrated by the functional interplay of *cis*-acting regulatory elements and *trans*-acting factors. The establishment of cell identity during development and the response to environmental cues such as pathogens requires transcriptional reprogramming (1, 2). Cells respond to signals by engaging various receptors that recognize and interpret the environment by initiating signal transduction cascades, culminating in the activation of transcription factors (TFs), which bind to specific regulatory DNA sequences to control the expression of genes, thus orchestrating a dynamic interplay between genome form and function (3–6). Inappropriate regulation of gene expression during development or adult life can cause abnormal embryogenesis and disease (7, 8). Enhancers and promoters represent the major classes of DNA regulatory elements responsible for warranting the execution of precise gene expression programs by functioning as information processing units interpreting the extra- and intra-cellular signals in the form of transcription factor binding events, which are followed by the recruitment of various transcriptional regulatory proteins such as cofactors, chromatin modifiers and the transcriptional apparatus at the core promoter (9–15). Core promoters are DNA regions, surrounding the start site of transcription (TSS), directing the accurate initiation of transcription by interacting with the various components of the general transcription machinery, including RNA Pol II (16). In addition, the DNA region (100-200 bp) located immediately upstream of the TSS often bears critical transcription factor binding sites and it is required to increase the rate of transcription (17, 18). In some cases, upstream promoter elements can function in a cell-type specific manner to mediate the interactions with distal regulatory elements like the enhancers (19, 20).

Enhancers activate transcription of target genes independent of their distance, position and orientation relative to the promoter of the affected gene (10, 21, 22). In certain cases, enhancers can act synergistically to ensure for proper gene activation (23). Our current model envisions that TFs recognize enhancer DNA in a combinatorial manner by reading the transcriptional regulatory code through the interplay of nucleotide sequence recognition, sequence context, 3D DNA structure (shape), DNA binding cooperativity, interacting cofactors and nucleosome occupancy (24–29). Establishment and maintenance of cell identity and function arise from integrated actions of transcription factors with chromatin–associated proteins, coactivators, and with the spatially organized genome (30). Combinatorial TF DNA binding leads to a synchronized targeting of enhancers by multifunctional transcriptional regulatory proteins such as coactivators, corepressors, chromatin modifiers and remodelers through direct and indirect physical associations, to assemble a plethora of high-order multi-protein-DNA and protein-protein complexes required for regulation of transcription (4, 9, 14). TFs belong to a diverse class of DNA binding protein families with distinct structural characteristics and individual members from the same family can bind similar DNA motifs, yet carry out distinct functions, *in vivo* (31–33). In addition, as the number of DNA binding sites in the genome for most TFs is greater than the number of the corresponding protein molecules per cell, it appears that only some TF molecules occupy most of their functional relevant sites in physiological conditions (34–38). TFs typically recognize and bind to short 6-12bp-long degenerate DNA motifs. Consistently, TFs bind to thousands of genomic sites across chromatin landscapes, but the number of genes changing their expression is frequently limited to a few hundred (39–42). Cells, at least in part, resolve this apparent paradox through combinatorial strategies where TFs must work together when bound to enhancers and promoters to achieve the specificity required for the selection of the appropriate genes to become activated. Cooperative DNA binding of TFs to low-affinity sites provides the means to target the correct genes for expression in time, space and in response to signals and is mediated by direct or indirect DNA-and/or chromatin-facilitating contacts between the proteins (33). Interestingly, high throughput ChIP-seq studies identified that binding of TFs to certain genomic regions is highly clustered, each cluster is composed of both functionally related and unrelated TFs, representing strong binding of one or a few TFs, and weaker binding to more degenerate motifs by many other TFs, implying a substantial degree of cooperation between individual motifs (43, 44). Large genomic regions with an unusually high degree of enrichment for lineage-specific TFs, cofactors (e.g. Med1) and histone modifications (H3K27ac) have been recently identified and characterized as super-enhancers (SEs), different from classical enhancers. These clusters of closely spaced enhancer elements often regulate the expression of genes determining cell identity and fate and driving oncogenic transcription (45–48). In general, super-enhancers are involved in strong intradomain interactions that is, between its individual enhancer constituents, which are stronger compared to typical enhancer-promoter interactions (15, 49). As super-enhancers consist of multiple enhancer elements, we assume that there will be complex functional relationships among the different constituents of any given super-enhancer working, depending on the biological context, either in a synergistic or additive manner or a combination thereof, thus resembling the arrangement and functional relationships of TF motifs building a typical enhancer.

One of the best-characterized examples of assembled super-enhancers is provided after stimulation of endothelial cells with TNFα, which leads to the formation of super-enhancers specific for inflammatory genes (50). Major players for the inducible assembly of these super-enhancers are the transcription factor NF-κB and the coactivator BRD4, a member of the bromodomain and extraterminal domain (BET) family of factors that is typically recruited to enhancers and super-enhancers. In brief, stimulation of endothelial cells with TNFα promotes the rapid redistribution of NF-κB across diverse genomic loci and subsequently the assembly of inflammatory SE chromatin configurations. This is accompanied by striking colocalization of the NF-κB p65 subunit and BRD4 in regulatory sequences marked by histone acetylation (H3K27ac). Thus, NF-κB efficiently targets its binding sites and promotes a pronounced recruitment of BRD4 across inflammatory SEs, which coordinate the development of a pro-inflammatory gene expression program (50).

The blood cell-specific SE cluster, known as BENC, is located 1.7 MB downstream of the *Myc* locus, and consists of multiple enhancer modules working in a combinatorial manner. BENC is bound by the transcription factors GFI1b, RUNX1 and MYB (51) and is essential for the regulation of *Myc* expression in hematopoietic and leukemic stem cells critical for hematopoietic malignancies. BENC deletion in hematopoietic stem cells closely mimics the conditional deletion of the *Myc* gene. Thus, overlapping, adjacent and/or partially redundant regulatory units arranged in clusters and bound by a variety of transcription factors functioning in a combinatorial manner, are essential for the generation of cell-type specific transcriptional regulatory outputs.

## Enhancer-Promoter Networks

The application of high-throughput sequencing-based chromatin profiling (e.g. ChIP-seq, DNAseI-seq), ideal for measuring the potential regulatory activity, indicates that the human genome contains about a million candidate different enhancers (52, 53) interspersed at regions located proximally, distantly, or within genes, and that in each cell type a fraction of these putative enhancers is active. Enhancers regulate gene expression in quite diverse manners. For example, enhancers do not necessarily regulate their closest gene (10), each gene may be regulated by more than one enhancer (54, 55), and a single enhancer can regulate multiple genes (56). The above mechanistic principles suggest the existence of complex and highly dynamic enhancer-promoter networks and diverse modes of enhancer action. The multiplicity of the enhancer-promoter functional interactions is highly regulated by transcription factors and cofactors associated with them to direct the re-configuration and the spatial folding of the 3D chromatin interaction, thus bridging remotely located transcriptional regulatory elements to a functional assembly. Although there is strong evidence that enhancer-promoter interactions are usually established concurrently with gene activation, and thus comprising an integral part of global genomic regulation, it is not yet proven whether enhancer-promoter contacts are the cause or the consequence of regulated gene expression. Some enhancer-promoter paired configurations are formed in the absence of any transcriptional events, thus suggesting that, simply, their identification does not necessarily have functional implications (57). However, forced chromatin looping between an enhancer and a promoter led to induction of transcription at high levels (58), thus strongly suggesting the requirement for establishing functional 3D structures connecting enhancers and promoters that could be integrated into a higher order condensed multi-genic 3D transcriptionally-active hubs at specific nuclear positions to coordinate gene expression of functionally related genes (59, 60).

Furthermore, although the identification of specific enhancer-promoter contacts provides important information regarding the assignment of enhancers to the correct target genes, these interactions are not necessarily predictive of functional regulation. For example, recent studies have shown that sonic hedgehog (Shh) expression in the developing embryo is regulated by multiple enhancer elements. Using a combination of 3D-DNA FISH and chromosome conformation capture approaches, Benabdallah et al., demonstrated that Shh expression is incompatible with the classic enhancer-promoter looping model. The authors found that, practically, there is no spatial proximity between the enhancer and the promoter during the differentiation of embryonic stem cells to progenitor neural cells (61). In contrast, however, Shh expression in the zone of polarized activity in the developing limb in mice, which is controlled by a different enhancer called ZRS, is characterized by the highest enhancer-promoter proximity as compared to other non-expressing tissues, a finding consistent with the formation of a tight chromosomal loop between the enhancer and the promoter (62). Similar to the findings of Shh expression in ES cells, live-cell imaging experiments revealed that the expression of the key pluripotency transcription factor Sox2 in ES cells is not controlled by the spatial proximity between its control region SCR and the promoter, despite the fact that 3C assays have identified enriched contacts between the enhancer and the promoter (63). Although it is not yet fully understood how a spatially remote enhancer could affect the activity of its target promoter, we speculate that the assembly of condensates composed of transcriptional regulatory proteins could trigger the formation of large macromolecular bridges or hubs, which may alter the configuration of the intervening chromatin structure, thus leading to increased enhancer-promoter spatial distances, despite the fact that these regulatory sequences are functionally linked.

Essential questions related to the formation of dense enhancer-promoter or enhancer-enhancer networks are how they are established, maintained, or dissolved. We speculate that it would be difficult or nearly impossible for any distal enhancer to locate its target promoter if the genome was largely homogeneously structured, thus lacking an internal higher order organization. We now know that, in general, the hierarchical organization of chromatin involves the A (euchromatin) and B (heterochromatin) compartments, with compartment B marked by a more compact chromatin packaging as compared to compartment A (64), which is characterized by increased chromatin accessibility and higher gene expression levels (65). Chromatin contacts within each compartment are specifically enriched, but contacts between genomic regions present in the A and B compartments are not favored. Each chromatin compartment is further substructured to additional hierarchical layers termed Topological Associated Domains (TADs) (66, 67), where regions within the same TAD tend to interact more frequently with each other than with regions belonging to different TADs. Thus, within TADs, numerous genomic interactions (loops) are formed connecting regulatory elements such as enhancers and promoters, with a significantly smaller number of interactions spanning across the boundaries of adjacent TADs. TAD borders are frequently demarcated by the binding of CTCF, a TF functioning as a chromatin organizer (68). TAD anchors are frequently bound by the CTCF-cohesin complex usually in a convergent motif orientation (66) through a loop extrusion process mediated by the cohesin ring until the process is blocked at CTCF sites arranged in a convergent orientation (69). Mutations at the TAD boundaries can lead to chromatin reconfiguration at the TAD borders causing enhancers present in one TAD to activate transcription of genes present in the neighboring TAD (70). More specifically, rearrangement of TAD boundaries at the WNT6/IHH/EPHA4/PAX3 locus caused an extensive rewiring of the interactions between enhancers and promoters leading to limb malformations (70). The functional insulatory role of CTCF in gene regulation was further investigated in a study where the disruption of CTCF binding at the TAD boundaries *via* CRISPR led to the aberrant interaction of a constitutive enhancer with the PDGFRA oncogene in glioma (71). Interestingly, removal of CTCF binding sites both at the boundary and within TADs at the Sox9-Kcnj2 locus led to fusion of neighboring TADs, which, however, did not result in major effects on developmental gene regulation (72). In contrast, however, inversion of CTCF binding sites within the protocadherin enhancer rewires the enhancer-promoter interactions and alters gene expression patterns (73). Taken together, the data presented above indicate that the precise role of chromatin topology in enhancer/promoter function is determined by the specific genomic context.

An important issue regarding the multiplicity of enhancer-promoter network of interactions relates to the mechanisms by which an enhancer selects the correct promoter for activation. While within TADs, the number of candidate promoter targets for a given enhancer is relatively limited, in many cases enhancers and their target promoters are not adjacent in the primary DNA sequence, but interrupted by one or more genes that are not co-expressed or co-regulated with the enhancer’s target gene. For example, expression of the Sex comps reduced (Scr) gene during Drosophila embryogenesis is controlled by an enhancer which is located far upstream. Importantly, the continuity of the Scr gene and its enhancer is interrupted by the localization of the fushi tarazu (ftz) gene, which is regulated by an enhancer positioned at close distance from the Scr gene, whereas the Scr enhancer is present at the 3’ of the ftz gene. In other words, the Scr enhancer is closer to ftz than to Scr promoter, and the ftz enhancer is flanked by the Scr and ftz promoters. However, both genes are expressed at different times during embryogenesis, and their expression is controlled by their individual enhancers. The selectivity of the Scr enhancer for activating transcription from the Scr promoter and not from the closest ftzp promoter depends on promoter proximal DNA elements called tethering elements, which when bound by specific transcription factors might mediate highly specific enhancer-promoter interactions between the Scr enhancer and promoter (19, 74). These data are compatible with the looping and relocation models for enhancer-promoter communication, because they can explain how an enhancer can skip more proximal promoters (75).

The structural chromatin loops responsible for forming TADs are dependent on ubiquitously expressed CTCF and cohesin proteins and therefore are distinct from those assembled by the interactions of regulatory sequences. The latter are generally mediated by enhancer- and promoter-bound transcription factors and cofactors and therefore their formation is dynamic and developmentally regulated (30). Our current understanding is that TADs define regions of the genome where enhancer-promoter interactions are allowed to occur to ensure robust and reliable transcriptional activation, thus precluding inappropriate regulatory interactions between enhancers and promoters located in different TADs (76). Despite the established roles of the architectural protein CTCF in supporting regulatory enhancer-promoter interactions (66, 73), its depletion although leads to a significant reduction of TAD structures results in moderate rather than dramatic changes in global gene expression (77, 78). An emerging idea derived from single-cell 3C and advanced imaging experiments (79, 80) to explain this unexpected finding is that the cell-to-cell variability in the overall TAD structure indicates that the existence of a highly heterogeneous structure of the chromatin could provide a reasonable explanation for the relatively modest effects in transcription upon TAD disruption. Thus, it is not yet fully understood how TADs and TAD boundaries affect the regulation of gene expression, and the regulatory logic that rules their involvement.

### Understanding the Regulatory Genome in 3D

The spatial arrangement of the regulatory genome in 3D has been mainly studied by Chromatin Conformation Capture (3C) methodologies and its ChIP or probe-based derivatives and advanced high-resolution microscopy techniques (62, 66, 81–85). Notably, some of the first examples of well-characterized mechanisms of gene regulation by DNA-DNA contacts in mammals, were derived from studies in hematopoietic and immune cells regarding the 3D organization of the β-globin gene locus (86) and the interchromosomal association between the *IFN-gamma* gene locus and regulatory regions of the T(H)2 cytokine locus in a dynamic and cell-type-specific fashion (87). Additional studies have provided evidence that transcription factors bound to enhancers and promoters mediate this type of DNA-DNA communication by acting as the components exposing complementary interacting protein surfaces required for the establishment and the specificity for the formation of looped structures (20). A well-characterized example of a looping factor essential for chromatin interactions at the β-globin locus is the cofactor Ldb1 (58). Ldb1 together with GATA1 acts as a molecular bridge, which facilitates the communication between the Locus Control Region (LCR) and the β-globin promoter.

**Figure 1:**
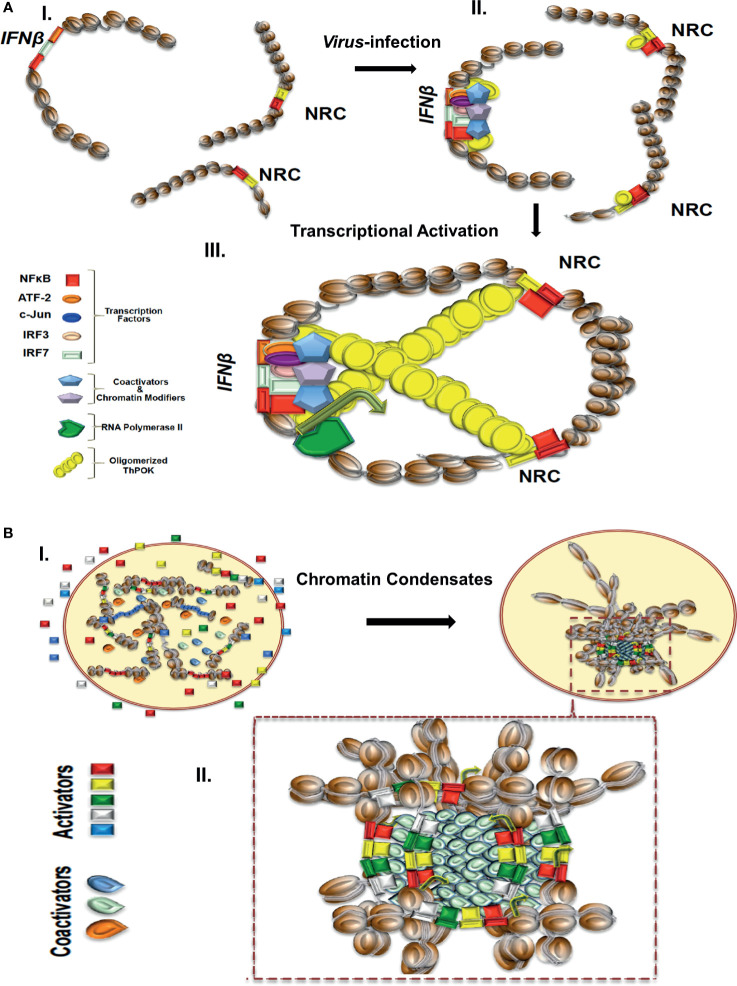
**(A)** A model depicting the virus infection-induced NRC-IFNβ interchromosomal interactions. In uninfected cells, the IFNB and NRC loci are well separated. Virus infection induces the nuclear localization of NF-κB, which binds cooperatively with ThPOK to appropriately spaced binding sites at NRCs (right part). While bound to NRCs, ThPOK oligomerizes to reach to a single *IFNβ* allele resulting in physical proximity of the NRCs-bound NF-κB with the *IFNβ* enhancer triggering enhanceosome assembly and subsequently activation of the *IFNβ* gene. **(B)** A model depicting the assembly of enhancer hubs and transcriptional condensates. In resting cells enhancers and transcriptional regulators are diffused in the nucleus. Upon cell activation transcriptional condensates bearing enhancers, promoters and regulators are formed as a result of liquid-liquid phase separation. Components are highly concentrated within the condensates.

In the case of the virus inducible human *IFNβ* stochastic gene expression, the IFNβ enhancer contributes to the formation of highly specialized 3D associations required for singular and stochastic expression of the *IFNβ* gene in a subset of infected cells (35, 88, 89). Previous work has indicated that virus infection induces the coordinated activation of three distinct sets of transcription factors, NF-κB, IRF3 and 7, and ATF2/cJun, which together with the architectural protein HMG I(Y) bind cooperatively to the nucleosome-free enhancer/promoter of the *IFNβ* gene and form a nucleoprotein structure known as the enhanceosome (24, 90–95) (**Figure 1**). Following its assembly, the IFNβ enhanceosome exposes a continuous recognition surface used to recruit the transcriptional coactivator CBP (93). Enhanceosomes with similar properties have now been identified and characterized on several model enhancers in both *Drosophila* and mammalian systems. For example, cell type-specific and/or inducible expression of the MHC class I and MHC class II expression, which is crucial for the initiation and regulation of adaptive immunity, requires the assembly of enhanceosomes which activate transcription by recruiting the coactivators NLRC5- and CIITA, respectively (96). As it is the case for the IFNβ enhanceosome, the NLRC5 and CIITA coactivators are recruited to their target promoters by interacting simultaneously with a continuous surface of the MHC Class I and Class II enhanceosome components, respectively.

The human *IFNβ* promoter contains one well-positioned nucleosome physically obscuring the TATA box and the start site of transcription. Once assembled, the IFNβ enhanceosome dynamically recruits histone acetyltransferases and chromatin remodelers causing sliding of the nucleosome masking the TATA to a new position 36bp downstream, thus unmasking the TATA box and the start site of transcription (91, 97, 98). The gene is initially expressed from a single allele, but its expression is subsequently becoming biallelic (88, 99). The discovery of the early monoallelic *IFNβ* expression suggested that gene activation itself is successfully completed only with a certain probability in a given cell. A similar mode of monoallelic stochastic activation has also been described for the *IFNα* family of genes and it is a typical and general feature of cytokine expression during immune responses (100, 101).

Given the pivotal role of *IFNs* in antiviral host responses, it has come as a surprise that, at the single-cell level, *IFNs* are expressed only by a fraction of virus-infected cells. Indeed, approximately ~20% of the virus-infected cells are expressing *IFNβ*. Moreover, the induction of Interferon Stimulated Genes (ISGs) after acute viral infections has also been found to be highly heterogeneous at the single-cell level (99), with only a fraction of cells inducing an antiviral gene expression program at physiological concentrations of IFNs.

Recent findings have indicated that IFNβ expression heterogeneity is due, at least in part, to stochastic interchromosomal interactions driving the variable patterns of *IFNβ* gene expression in response to virus infection (35, 88, 89). The important finding was that stochasticity in expression of human *IFNβ* is due to low intra-cellular concentrations of the transcription factor NF-κB, which is captured onto specialized DNA sites called NRCs (NF-κB Reception Centers) belonging to Alu-like repetitive elements and then it is delivered to the IFNβ enhancer, *via* stochastic interchromosomal interactions. It was shown that the transcription factor ThPOK (89), a GAGA binding factor, binds cooperatively with NF-κB to NRCs and mediates their physical proximity with the *IFNβ* gene and other co-regulated genes *via* its ability to oligomerize when bound to DNA, as it was shown by *in vitro* experiments. Furthermore, ThPOK knockdown experiments significantly decreased the frequency of interchromosomal interactions, NF-κB DNA binding to the IFNβ enhancer, and virus-induced *IFNβ* gene activation. Cooperative DNA binding between ThPOK and NF-κB, *in vitro* and *in vivo*, on the same face of the double DNA helix, is required for interchromosomal interactions and this distinguishes NRCs from various other Alu elements bearing interspersed NF-κB and ThPOK sites (89) (**Figure 1A**). These studies showed how DNA binding cooperativity of stereospecifically aligned transcription factors provides the necessary ultrasensitivity for the all-or-none stochastic cell responses to virus infection. The above mechanistic insights strongly suggest that the primary DNA sequence of the interacting DNA elements acts as a blueprint, which contains a specific linear genomic code that is interpreted *via* the construction of highly specialized high-order nucleoprotein complexes. Additional studies have also shown that stochastic *IFNβ* expression in response to virus infection is due to cell-to-cell variability due to limiting quantities of components ranging from the recognition of viral RNA by host factors and the activation of signaling pathways, to the exact levels of activated transcription factors (102).

Additional examples of architectural proteins acting as organizers of 3D genome architecture include YY1 (103), ZNF143 (104), and SATB1 (105). In contrast to CTCF, YY1 preferentially binds to active enhancers and promoters mediating their 3D interaction *via* its ability to dimerize (103). ZNF143 binds to the anchors of chromatin interactions and facilitates the formation of secure connections between promoters and distal regulatory elements (104). Finally, SATB1 can act as a tissue-specific organizer of gene expression with well-established roles in the regulation of TH2 cytokine gene locus (105, 106) by cooperative binding to multiple closely spaced consensus sites in nucleosomes (107). In summary, the examples described above demonstrate that the formation of 3D DNA-DNA nuclear contacts is not a purely random phenomenon but it depends on sequences hosting chromatin architectural proteins, which in many cases are in direct interaction with TFs.

The molecular mechanisms controlling olfactory receptor (OR) gene transcription (108) inducing the monoallelic expression of one out of more than 1,000 genes in each cell, share many common features with the cellular responses leading to virus-induced stochastic monoallelic *IFNβ* expression. The singular and stochastic expression of OR genes is controlled by a dense network of 3D DNA interactions occurring exclusively in olfactory sensory neurons (109–111). The OR genes, which are interspersed in various chromosomes, aggregate in one nuclear compartment, where they are repressed by acquiring chromatin marks characteristic of heterochromatin. However, this aggregation brings in close proximity many OR enhancers located adjacent to the OR genes *via* multiple interchromosomal interactions forming a multi-enhancer hub. Monoallelic OR transcription is activated by the multi-enhancer hub, which associates stochastically with the corresponding gene chosen for expression. The TFs Lhx2 and Ebf bind cooperatively in a stereospecific manner on OR enhancers followed by the recruitment of the LIM domain protein Ldb1, which facilitates these interchromosomal interactions. Thus, DNA binding cooperativity between transcription factors, as it is also the case for *IFNβ* expression, provides the necessary specificity to drive spatiotemporal alterations of the 3D chromatin for the cell-type specific expression of OR genes and cellular responses upon viral infections, respectively.

Many mechanistic similarities to the stochastic monoallelic OR and *IFNβ* expression are also shared by the NF-κB-dependent TNFα-induced hierarchical co-expression of *SAMD4A*, *TNFAIP2*, and *SLC6A5* genes (112, 113), which also form a tight signal-dependent multigene complex through intra- and interchromosomal interactions. TNFα induction causes the hierarchical co-transcription of these genes in a small fraction of cells containing tightly associated genes in a multigene complex.

Photoreceptor specification in the Drosophila eye depends on the stochastic and cell-autonomous expression of the transcription factor Spineless (Ss) (114–116). The stochastic *Ss* expression in individual cells is regulated by a 60 kb region harboring the R7/8 enhancer and the silencer elements 1 and 2 (115, 116). It was shown that the stochastic Ss expression pattern depends on interchromosomal communication and on inter-allelic crosstalk, which averages the frequency of expression of each allele (116). Thus, although each allele follows an independent-stochastic choice of expression, 3D interchromosomal communication coordinates the expression status between alleles, thus ensuring for the proper expression of both alleles within the same randomly selected subset of photoreceptor cells. Conclusively, both the 3D interchromosomal interactions and the action of enhancers from a distance determine stochastic photoreceptor specification.

In summary, the examples described above when taken together with many other similar studies, suggest a general model according to which cell activation by environmental signals or during development and differentiation leads to a cascade of multiprotein and multigene high order dynamic assemblies, ranging from signalsomes to enhanceosomes and chromatin-modifying machines, thus providing local regulatory input from different genomic elements. This mode of information flow ensures that stochastic molecular interactions are canalized to produce specific, robust and reliable outputs. Furthermore, these observations strongly support the view that transcription factors are capable of inducing extensive chromatin rewiring, in many cases stochastic, at specific genomic loci, thus modifying gene expression patterns in a cell-specific manner.

## Enhancer Hubs-Transcriptional Condensates

Enhancer hubs containing highly interacting regulatory elements represent a remarkably dynamic and heterogeneous network of multivalent macromolecular interactions. Most of these interactions could be established in a stochastic manner and individual cells within a homogeneous population may contain unique combinations of two or more interacting partners. The higher the number of interacting partners the lower the probability of a cell in the population to contain multi-partner hubs. We speculate that enhancer hubs by containing multiple enhancers targeting a single promoter or many promoters targeted by a single enhancer or many interconnecting enhancers targeting many different promoters, could aggregate into a single nuclear position. A functional consequence of such complex interaction networks could be that different enhancers function synergistically to ensure coordination, tight control and robustness in regulation of expression of spatially connected genes. Thus, enhancer-promoter hubs ensure that all target genes are “on” and are appropriately regulated in a spatiotemporal manner, with stable relative levels of expression. Thus, each gene is subject to the same intrinsic and extrinsic noise e.g. fluctuations in the concentration of transcriptional regulators etc. The high density of transcription factors bound to enhancers and promoters in the context of enhancer hubs or super-enhancers leads to the cooperative and synergistic recruitment of high concentration of coactivators, components of the transcriptional machinery and other cofactors (117). Conclusively, an important implication derived from the assembly of enhancer hubs is the accumulation of transcriptional regulators at high local concentrations, which then are equally available for binding to the enhancer and promoter elements of the associated genes in a locus-specific manner.

Since many transcriptional regulators typically contain large low complexity and/or intrinsically disordered regions (IDRs), like the activation domains of many TFs, the probability to undergo hetero- or homo-typic multivalent interaction or oligomerization is high (118). IDRs have been described as functional protein segments that are not likely to form a defined 3D structure (119) and have been classified mainly by their amino acid profile or by their hypothetical shape (120). High densities of proteins bearing IDRs and nucleic acids have been implicated in the formation of membraneless compartments through the well-understood mechanism of liquid-liquid phase separation (LLPS) (121–125). The term phase separation describes a process by which distinct liquid droplets bearing high concentration of molecules are formed. The formation of such nuclear compartments increases the likelihood for certain biochemical reactions to occur, such as transcription, thus bypassing partially or entirely the necessity for direct physical contacts between individual enhancers and promoters (**Figure 1B**). Theoretically, an additional advantage of regulating coordination of transcription *via* a phase separation model is the plasticity and reversibility by which LLPS formations are driven, and thus provide a reliable mechanism of transcriptional initiation and termination of many coregulated genes at once. For example, clusters of enhancer elements have been proposed to regulate biological processes implicated in development and disease (47), as well as regulating mammalian cell identity (45). We speculate that in the case of the SEs assembling upon TNFα induction of endothelial cells (see above), the pronounced recruitment of cofactors, like BRD4, etc. could lead to the additional recruitment of RNA Pol II and cofactors *via* an extensive network of intermolecular interactions between specific regions of intrinsically disordered hydrophilic activation domains (IDRs), thus leading to the formation of phase-separated nuclear liquid droplets (126) (**Figure 1B**).

The proposed model describes that the highly condensed concentration of biochemical interactions between TFs and coactivators, occurring on TFBSs-enriched extended 3D chromatin configurations, promote the colocalization/co-recruitment of diverse SEs at specific nuclear sites and subsequently the formation of 3D chromatin condensates (**Figure 1B**).

The ability of eukaryotic cells to assemble high-order transcription condensates ensures that enhancers bearing low-affinity binding sites can efficiently recruit RNA Pol II molecules to target genes to support robust patterns of gene expression (27, 125, 126). It is reasonable to assume that the individual functions of SEs could become modified upon their recruitment to the 3D chromatin condensates, where they could acquire novel regulatory characteristics hardwired in dense networks of macromolecular interactions.

## Author Contributions

All authors contributed to the article and approved the submitted version.

## Funding

DT was supported from the Greek Secretariat for Research and Technology (Cooperative Grants SYNERGASIA I #969, Excellence Award ARISTEIA I #1567, European Committee (FP7 projects INTEGER, NANOMA, PREDICTA and BIOFOS), from the European Economic Area (EL0084), BIO-IMAGING GR (MIS-5002755), and from the KMW offsets program. MA was supported from research grants from the Bodossaki Foundation, Fondation Santé, Gilead Hellas, and Antonios and Ioannis Angelicoussis Foundation. The funder was not involved in the study design, collection, analysis, interpretation of data, the writing of this article or the decision to submit it for publication.

## Conflict of Interest

The authors declare that the research was conducted in the absence of any commercial or financial relationships that could be construed as a potential conflict of interest.
